# Spontaneous combined rupture of a pelvicalyceal cyst into the collector system and retroperitoneal space during the acquisition of computed tomography scan images: a case report

**DOI:** 10.1186/1752-1947-6-386

**Published:** 2012-11-13

**Authors:** Diogo Torres Marques, Regis Otaviano Franca Bezerra, Luiz Tenório de Brito Siqueira, Marcos Roberto Menezes, Manoel de Souza Rocha, Giovanni Guido Cerri

**Affiliations:** 1 Department of Radiology, Sírio Libanês Hospital, Adma Jafet 91, Bela Vista, SP, Zip Code 01308-050, Brazil

**Keywords:** Collector system, Computed tomography pelvicalyceal cyst, Renal lithiasis, Retroperitoneum, Spontaneous rupture

## Abstract

**Introduction:**

Pelvicalyceal cysts are common findings in autopsies and can manifest with a variety of patterns. These cystic lesions are usually a benign entity with no clinical significance unless they enlarge enough to cause compression of the adjacent collecting system and consequently obstructive uropathy. Few cases of the spontaneous rupture of pelvicalyceal renal cysts have been published and to the best of our knowledge there is no report of a combined rupture to collector system and retroperitoneal space documented during a multiphase computed tomography.

**Case presentation:**

We report a case of a ‘real-time’ spontaneous rupture of a pelvicalyceal cyst into the collecting system with fistulization into the retroperitoneum.

The patient was a 78-year-old Caucasian man with a previous history of renal stones and a large pelvicalyceal renal cyst who was admitted to our Emergency department with acute right flank pain.

A multiphase computed tomography was performed and the pre-contrast images demonstrated a right pelvicalyceal renal cyst measuring 12.0 × 6.1cm in the lower pole causing moderate dilation of the upper right renal collection system. In addition, a partially obstructive stone on the left distal ureter with mild left hydronephrosis was noted.

The nephrographic phase did not add any new information. The excretory phase (10-minute delay) demonstrated a spontaneous rupture of the cyst into the pelvicalyceal system with posterior fistulization into the retroperitoneal space.

**Conclusion:**

In this case study we present time-related changes of a rare pelvicalyceal cyst complication, which to the best of our knowledge has fortunately not been previously documented. Analysis of the sequential images and comparison with an earlier scan allowed us to better understand the physiopathological process of the rupture, the clinical presentation and to elaborate hypotheses for its etiopathogenesis.

## Introduction

Pelvicalyceal cysts are common findings in autopsy cases and can manifest with a variety of patterns from multiple small cysts to a large solitary lesion [1,2]. Their etiology is not very well defined but they are believed to be of lymphatic origin and no hereditary pattern has been shown [2].

On radiologic images the pelvicalyceal cysts appear as rounded or oval lesions with water attenuation replacing the renal sinus fat and can be difficult to differentiate from hydronephrosis on unenhanced computed tomography (CT), magnetic resonance imaging (MRI) or sonography [3]. The use of contrast-enhanced CT or MRI scan or excretory urography enables this differentiation by demonstrating the renal pelvis filled with contrast medium and dislocated or compressed by the water-attenuation cyst which has no communication with the collecting system and therefore is without contrast in its interior. These examinations are also useful to differentiate renal sinus cysts from calyceal diverticulum, because the last has a narrow passage to the collecting system and will also be filled with contrast medium [4].

These cystic lesions are usually a benign entity with no clinical significance unless they enlarge enough to cause compression of the adjacent collecting system and consequently obstructive uropathy [2,3]. Spontaneous rupture is a rare event that has been reported in few cases in which there is drainage to the pelvicalyceal system, the perirenal space or the peritoneal cavity [5-8]. When such complication happens it can lead to symptoms such as abdominal pain or hematuria.

## Case presentation

**Figure 1:**
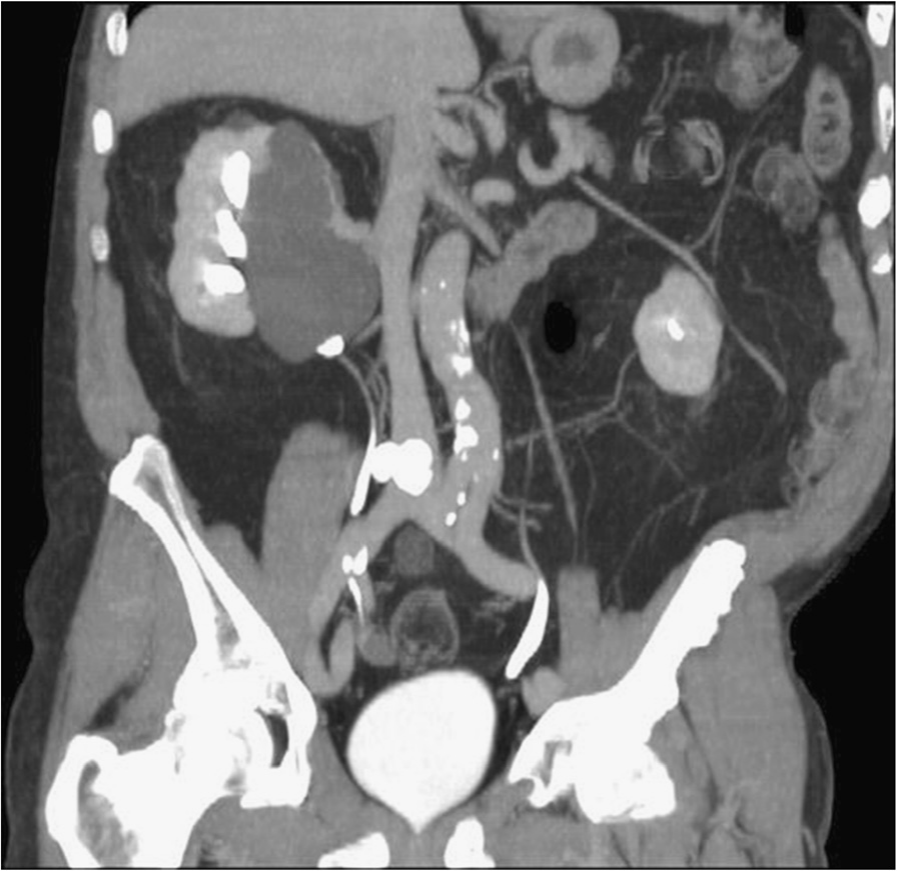
**Four months older computed tomography scan (coronal maximum intension projection reconstruction of the excretory phase).** This previous computed tomography scan demonstrates a large non-complicated pelvicalyceal cyst on the right kidney with no signs of communication with the collector system since it is not filled with contrast media.

A 78-year-old Caucasian man with acute flank pain on the right side and a history of bilateral renal stones was referred to our Emergency department. The patient denied any episode of fever or trauma. He had a multiphase CT scan taken 4 months earlier that showed a large homogeneous, thin-walled pelvicalyceal cyst on the right kidney measuring 10.0 × 5.7cm, tapering the ipsilateral renal pelvis, with no signs of communication with the collector system (Figure 1) or hydronephrosis. This previous CT also demonstrated a radiopaque calculus measuring 0.4cm located on the medium calyceal system of the left kidney.

**Figure 2:**
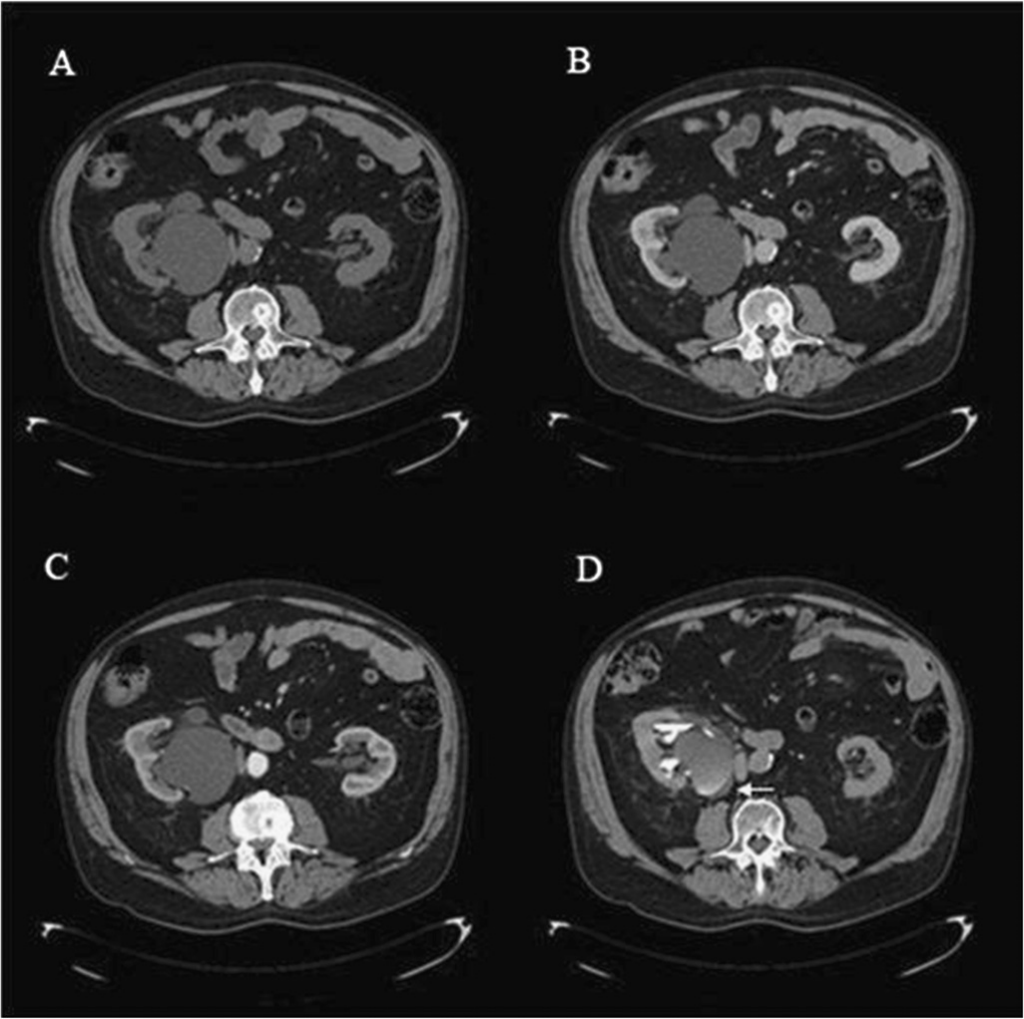
**Pre-contrast (A), venous (B), arterial (C) and excretory (D) phases of the current computed tomography scan.** Note the evolution of the cyst during the acquisition of the multiphase computed tomography images. The excretory phase **(D)** demonstrates that the cyst is smaller than the other phases (**A, B** and **C**), there is contrast inside and free fluid around (white arrow), also not present on the previous aquisitions.

**Figure 3:**
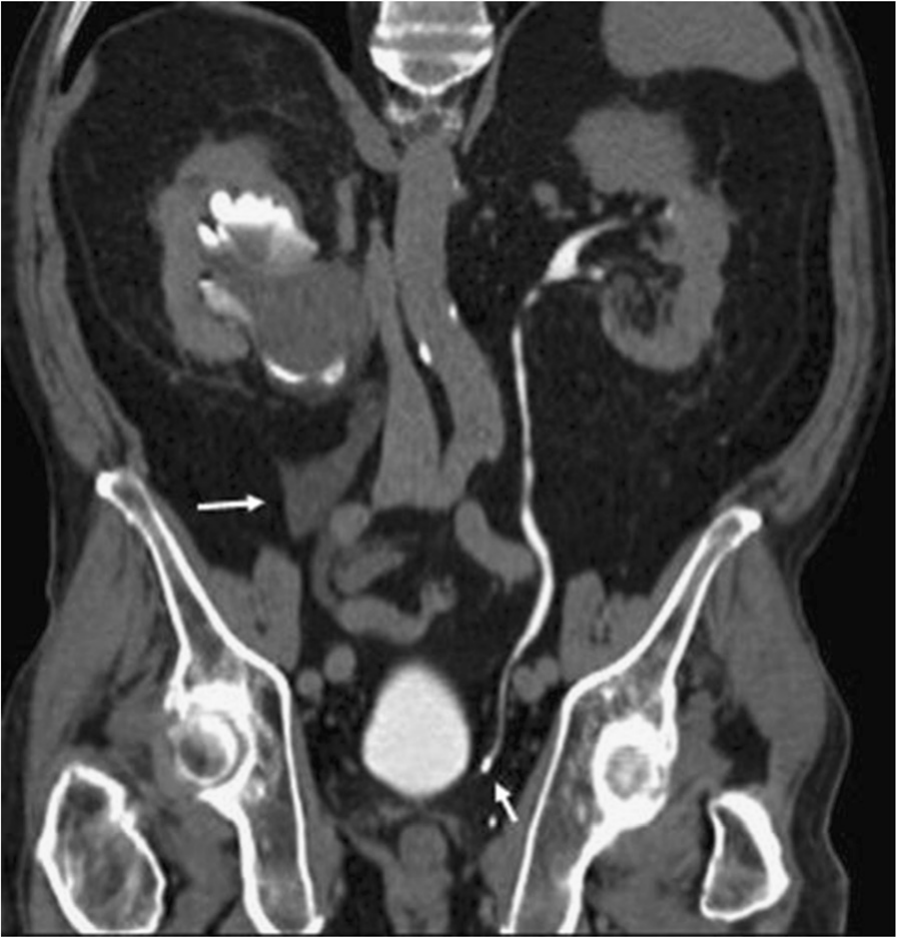
**Coronal maximum intension projection reconstruction from excretory phase of the current computed tomography scan.** This image shows the cyst with contrast inside, free fluid on the retroperitoneal space (big arrow) and the stone obstructing the left ureter (small arrow).

A new multiphase CT scan was requested by the emergency physician for further evaluation. The pre-contrast images revealed the pelvicalyceal cyst, now measuring 12.0 × 6.1cm, in the right lower renal pole abducting the ureteropelvic junction, and moderate right hydronephrosis. In addition, the stone was impacted on the contralateral distal ureter causing mild dilatation of the ipsilateral collector system. No free fluid was seen on the abdominal cavity and no other new information was obtained with the arterial and venous phases. Surprisingly, in the excretory phase the cyst presented with irregular contours, partially filled with radiopaque contrast material, and a small amount of fluid on the retroperitoneal space was noted (Figures 2 and 3).

These findings demonstrate a spontaneous rupture between the renal cyst and the pelvicalyceal system with, consequently, fistulization into the retroperitoneal space occurring between venous and excretory phases.

**Figure 4:**
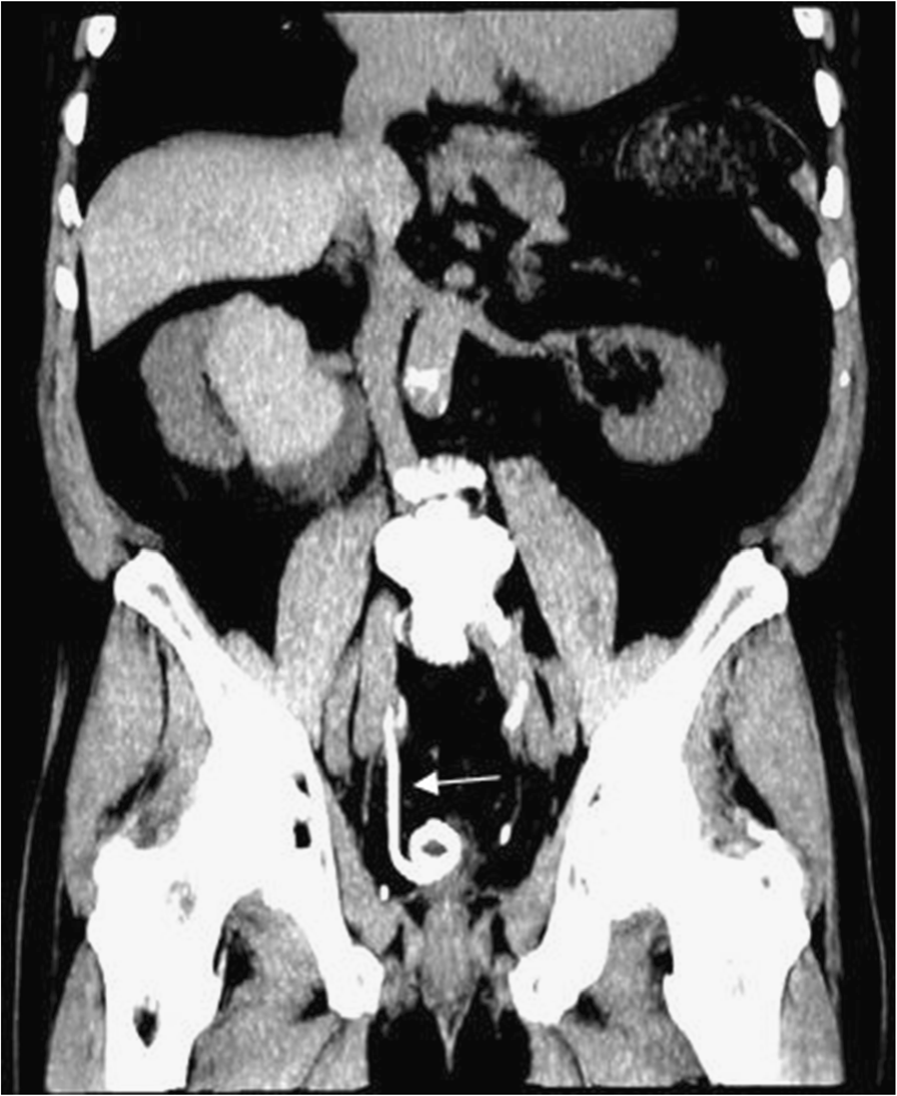
**Four days later computed tomography scan.** This computed tomography scan obtained 4 days after the rupture shows the cyst with residual contrast, confirming the communication with the pelvicalyceal system, and the placement of the double-J catheter for treatment (arrow).

The follow-up CT scan, obtained 4 days later, confirmed the communication of the cyst with the pelvicalyceal system and the patient was treated with a retrograde double-J catheter placement (Figure 4). Although, the stone was still impacted in the left distal ureter with mild hydronephrosis, the patient was completely asymptomatic.

## Discussion

To the best of our knowledge, there is no report of the spontaneous rupture of a pelvicalyceal cyst into the collecting system with posterior fistulization into the retroperitoneal space documented during a multiphase CT scan acquisition. The patient in this case also has a contralateral ureteral stone that might contribute to an increase in pressure in the right kidney.

In addition, the comparison with the 4 months older CT documented a slight increase in the size of the cyst, which probably collaborated for the final outcome.

A combination of hypotheses would explain all the image findings of this patient:

1. Increase of intrarenal pressure by administration of endovenous contrast. The presence of contrast media particles inside renal tubules, which are not absorbed by renal cells, causes an osmotic effect leading to a marked increase of water and sodium excretion [9]. This induced diuresis will consequently elevate intrapelvic pressure.

2. Elevation of filtration rate and urine flow by the right kidney to compensate the obstruction of the left collector system caused by the urolithiasis [10].

3. The enlargement of the pelvicalyceal cyst may cause progressive compression in the collector system and consequently high pressures.

## Conclusion

In this case study we present time-related changes of a rare pelvicalyceal cyst complication, which to the best of our knowledge has fortunately not been previously documented. Analysis of the sequential images and comparison with an earlier scan allowed us to better understand the physiopathological process of the rupture, and to raise three hypotheses for its etiopathogenesis.

## Consent

Written informed consent was obtained from the patient for publication of this case report and accompanying images. A copy of the written consent is available for review by the Editor-in-Chief of this journal.

## Competing interests

The authors declare that they have no competing interests.

## Authors’ contributions

ROFB and LTBS were the radiologists who first identified the case during their routine reports. Together with DTM the data were collected and the article elaborated. MRM, MSR and GGC helped with the revision of the report, also adding new information to the discussion topic. All authors read and approved the final manuscript.
